# Non‐visualized Ultrasonography Should Remain Indeterminate in Patency Capsule Localization

**DOI:** 10.1002/deo2.70344

**Published:** 2026-06-27

**Authors:** Anastasios Koulaouzidis, Ervin Toth

**Affiliations:** ^1^ Department of Clinical Research University of Southern Denmark (SDU) Odense Denmark; ^2^ Department of Surgery SATC‐C, Odense University Hospital and Svendborg Hospital Svendborg Denmark; ^3^ Department of Gastroenterology Pomeranian Medical University Szczecin Poland; ^4^ Department of Gastroenterology Skåne University Hospital, Lund University Malmö Sweden

To the Editor:

We read with considerable interest the prospective study by Matsuura et al. [1] evaluating abdominal ultrasonography (AUS) against computed tomography (CT) for patency capsule (PC) localization before small‐bowel (SB) capsule endoscopy. The clinical question is important, and the appeal of a radiation‐free first‐line strategy is obvious. Nevertheless, we believe the manuscript's central message is more confident than the data justify.

Our concern is the treatment of non‐visualized examinations. In the primary binary analysis, AUS findings reported as “colon” and “not visualized” were both counted as test‐negative [1]. This is the pivotal methodological step behind the paper's apparently perfect performance. However, a non‐visualized examination is not a true negative result; it is, clinically, an indeterminate one. Indeed, the authors themselves acknowledge that non‐visualization does not exclude SB retention and may still require CT [1]. To convert diagnostic incompleteness into a negative test risks overstating the real‐world capability of AUS [2, 3]. In practical terms, AUS provided definitive localization in 28/34 patients (82.4%), whereas 6/34 (17.6%) remained non‐diagnostic and still required CT [1]. Albeit useful, it does not amount to a definitive stand‐alone test. Further restraint is warranted because the reported 100% sensitivity for SB retention rests on only six CT‐confirmed events, with a wide 95% confidence interval of 61.0%–100% [1]. In addition, the AUS operator had access to prior X‐ray findings and the estimated PC location before scanning, introducing review bias [2, 3]. Finally, this was a single‐center expert study without interobserver agreement analysis, limiting generalizability [1, 4]. AUS appears promising as a triage tool, but non‐visualized examinations should remain indeterminate, not negative. Multicenter validation with blinded assessment is now needed.

## Author Contributions

**Anastasios Koulaouzidis** conceived the commentary, performed the methodological and clinical appraisal, interpreted the implications for WCE AI validation, and wrote the manuscript. **Ervin Toth** contributed to the writing and approved the final version.

## Funding

The authors have nothing to report.

## Conflicts of Interest

Anastasios Koulaouzidis and Ervin Toth are or have been Guest editors for MDPI; Anastasios Koulaouzidis and Ervin Toth are Section board members of *Diagnostics* and JCM. Anastasios Koulaouzidis is Associate Editor of the *Annals of Gastroenterology, Diagnostics* and *Therapeutic Advances of Gastrointestinal Endoscopy*. Anastasios Koulaouzidis is the immediate past president of the iCARE group.

**Disclosure(s)**: Anastasios Koulaouzidis is a co‐founder shareholder of AJM Med‐i‐caps Ltd and a consultant for Jinshan Science & Technology Ltd. He has also been co‐founder and director of iCERV Ltd. Anastasios Koulaouzidis received research support in the form of grants from Given Imaging Ltd under the auspices of the European Society of Gastrointestinal Endoscopy (ESGE), and from IntroMedic (also noted as SynMed/Intromedic), and has served as a consultant for Jinshan Ltd, with consultancy fees from Jinshan and lecture honoraria from Covidien/Medtronic. Additionally, he has benefited from lecture honoraria and educational travel support from a range of entities, including Jinshan, Dr Falk Pharma UK, Ferring, Aquilant, and Almirall, and has participated in advisory board activities for companies such as Tillots, Ankon, and Dr Falk Pharma UK. Ervin Toth has received research support from AnX Robotica; research support and lecture honoraria from Jinshan Science & Technology Ltd. and Norgine; consultancy and lecture fees from Medtronic and Olympus.

**Disclosure (AI‐in‐writing)**: Artificial intelligence tools (ChatGPT, OpenAI, San Francisco, CA, USA) were used solely to assist with language editing and style refinement during manuscript preparation. No AI‐generated data or figures were included. All scientific content, interpretations, and conclusions were developed, verified, and approved entirely by the authors.

The views and opinions expressed herein are personal to the authors and do not represent the official positions of any institutions, professional societies, or organizations with which the authors are affiliated or hold leadership roles.
